# Therapeutic Antitumor Efficacy of Cancer Stem Cell-Derived DRibble Vaccine on Colorectal Carcinoma

**DOI:** 10.7150/ijms.61510

**Published:** 2021-07-23

**Authors:** Changhao Fu, Geer Tian, Jinyue Duan, Kun Liu, Chen Zhang, Weiqun Yan, Yi Wang

**Affiliations:** 1 Department of Regenerative Medicine, School of Pharmaceutical Sciences, Jilin University, Changchun, Jilin 130021, China; 2 Stanford University Medical School, VA Palo Alto Health Care System, Palo Alto, CA 94304, USA,; 3 Institute of Oceanography, Minjiang University, Fuzhou, Fujian 350108, China; 4 Medical Institute of Regeneration Sciences, Jilin University, Changchun, Jilin 130021, China.

**Keywords:** DRips-Containing Blebs (DRibbles), Dendritic Cells, Cancer Stem Cells, Autophagosome, Colorectal Cancer

## Abstract

Dendritic cell (DC)-based immunotherapy has been a promising strategy for colon cancer therapy, but the efficacy of dendritic cell vaccines is in part limited by immunogenicity of loaded antigens. In this study, we aimed to identify a putative tumor antigen that can generate or enhance anti-tumor immune responses against colon cancer. CD44^+^ colon cancer stem cells (CCSCs) were isolated from mouse colorectal carcinoma CT-26 cell cultures and induced to form defective ribosomal products-containing autophagosome-rich blebs (DRibbles) by treatment with rapamycin, bortezomib, and ammonium chloride. DRibbles were characterized by western blot and transmission electron microscopy. DCs generated from the mice bone marrow monocytes were cocultured with DRibbles, then surface markers of DCs were analyzed by flow cytometry. Meanwhile, the efficacy of DRibble-DCs was examined *in vivo*. Our results showed that CCSC-derived DRibbles upregulated CD80, CD86, major histocompatibility complex (MHC)-I, and MHC-II on DCs and induced proliferation of mouse splenic lymphocytes and CD8^+^ T cells. In a model of colorectal carcinoma using BALB/c mice with robust tumor growth and mortality, DC vaccine pulsed with CCSC-derived DRibbles suppressed tumor growth and extended survival. A lactate dehydrogenase test indicated a strong cytolytic activity of cytotoxic T-cells derived from mice vaccinated with CCSC-derived DRibbles against CT-26 cells. Furthermore, flow cytometry analyses showed that the percentages of IFN-γ-producing CD8^+^ T-cells were increased in SD-DC group compare with the other groups. These findings provide a rationale for novel immunotherapeutic anti-tumor approaches based on DRibbles derived from colon cancer stem cells.

## Introduction

According to the last statistics of the International Agency for Research on Cancer, colorectal carcinoma is the third most frequent malignancies worldwide with an incidence of 1.85 million new cases per year and accounts for 10.2% of total malignancies 1. High mortality is due to the limited efficacy of traditional surgery and chemotherapy, accompanied by severe side-effects, high risk of recurrence, and acquiring of chemoresistance 2. Emerging research shows that there is a subpopulation of tumor cells within cancer cells, named cancer stem cells (CSCs) 3. They have distinct immunophenotypes and can self-renew and differentiate into heterogeneous lineages of cancer cells, which are responsible for traditional therapy failure, tumor relapse, and metastasis 4.

In the past two decades, dendritic cell (DC)-based immunotherapy has been extensively investigated and represents a promising therapeutic approach against cancer 5. DCs are the largest part of and the most effective antigen-presenting cells in the immune system that can capture, process, and present extra- and intracellular antigens to professional T cells in complex with major histocompatibility complex (MHC) molecules 6. Several studies have shown that DC vaccines pulsed with tumor cell lysates or RNAs induced effective antitumor responses *in vitro* and *in vivo* 7-9. Our previous research had demonstrated that lysates of CD44^+^ CSCs derived from CT-26 colon cancer cell lines may be a possible source of tumor antigens to pulse DCs 10, in that they evoked potent tumor-specific CTL activity against colon cancer model* in vivo* and killed the CT-26 cancer stem cells* in vitro*. In order to improve the efficacy of cross-presentation of tumor-derived antigens and to enhance the T cell's anti-tumor immune responses, we focused on looking for a protein source containing a broader repertoire of tumor-associated antigens (TAAs).

Autophagy is a conservative cellular process of self-degradation in which damaged organelles or dysfunctional cellular components are surrounded by double-membrane vesical called autophagosomes and delivered to lysosomes for degradation and recycle 11. In tumor cells, TAAs are degraded by two major proteolysis pathways. The long-lived proteins are degraded through the autophagy-lysosome pathway 12, 13, whereas short-lived proteins and defective ribosomal products (DRiPs) are degraded by the ubiquitin-proteasome system 14. It is generally believed that short-lived proteins expressed by tumor cells are not cross-presented by host antigen-presenting cells, however, they are efficiently cross-presented when proteasome activity of tumor cells was inhibited 15. Therefore, with the induction of autophagy and inhibition of lysosomal/proteasomal activity, a broader repertoire of tumor antigens comprising long-lived proteins, short-lived proteins, and defective ribosomal products (DRiPs) are sequestered in autophagosomes, named as DRiPs-containing autophagosome-rich blebs (DRibbles) 15. DRibbles are efficient carriers to ferry a broad spectrum of tumor antigens including heat shock protein (HSP) 90, HSP94, calreticulin, and HMGB1, which are critical in augmenting cross-presentation of antigens and triggering immunogenic responses 15-17. It was reported that the induction of autophagy enhanced the cross-presentation of these short-lived proteins and generated CD8^+^ T cells and kill tumor cells with higher efficiency. DC vaccines based on DRibbles from tumor cells were found to be highly immunogenic in the mice model 16-19.

To the best of our knowledge, the potential antitumor activities of DCs pulsed with colon CSCs (CCSCs)-derived DRibbles have not been previously investigated using a colon cancer model. CCSCs may contain distinct antigen profiles and T cells specific to CCSC-derived DRibbles that can target the subpopulation of CSCs in CT-26 cells and tumor tissue, and therefore inhibiting tumor growth. In this study, we aimed to evaluate the antitumor effects of DC vaccines prepared by pulsing DCs with CCSCs-derived DRibbles isolated from mouse colon carcinoma CT-26 cells using a BALB/c murine model of colon carcinoma.

## Materials and methods

### Mice and cell lines

Female BALB/c mice (6-8 weeks old) were purchased from Beijing HFK Bioscience Co., Ltd. and housed in the Laboratory Animal Center at Jilin University (Changchun, China). The mouse colon carcinoma CT-26 cell line was purchased from The Cell Bank of Type Culture Collection of the Chinese Academy of Sciences, and cultured in Roswell Park Memorial Institute 1640 (RPMI 1640; Gibco; Thermo Fisher Scientific, Waltham, MA, USA) supplemented with 10% fetal bovine serum (FBS; Gibco) at 37°C and 5% CO_2_.

### Magnetic-activated cell sorting (MACS)

Colon CSCs were magnetically separated from CT-26 cells (1 × 10^7^ cells/ml) after incubation with the biotin-conjugated anti-CD44 monoclonal antibody (Miltenyi Biotec, Bergisch Gladbach, Germany) using a CELLection Biotin Binder kit (Thermo Fisher Scientific) as previously described 10. CD44^+^ CT-26 cells were cultured in Dulbecco's modified Eagle medium/F12 (DMEM/F12; Gibco), supplemented with 20 ng/ml recombinant murine fibroblast growth factor (rmFGF), 20 ng/ml recombinant murine epidermal growth factor (rmEGF; both from PeproTech, Rocky Hills, NJ, USA), 2% B27 (Gibco), and 8 mM HEPES (HyClone; GE Healthcare Life Science, Logan, UT, USA) at 37°C with 5% CO_2_. CD8^+^ T cells were purified from mouse splenocytes using EasySep Mouse CD8a Positive Selection Kit II (STEMCELL Technologies Inc. Vancouver, BC, Canada) according to the manufacturer's protocols.

### Tumorsphere-formation assay

Single-cell suspension of sorted CD44^+^ CT-26 cells was seeded on uncoated 96-well culture plates (Corning) at a density of 0.5 cells per 100 μl in each well, with fresh medium added every 3 days. Tumorsphere formation was observed for 14 days and images representative of at least five random fields were captured using an inverted light microscope (Olympus Corporation, Tokyo, Japan).

### Serum-induced differentiation

Undigested CD44^+^ CT-26 (5 × 10^5^) cell clumps were incubated for 5 days in RPMI 1640 medium supplemented with 10% FBS at 37°C with 5% CO_2_. Images of CD44^+^ CT-26 cells were acquired using an inverted light microscope (Olympus Corporation).

### Isolation of bone marrow-derived DCs

DCs were isolated from mouse bone marrow as previously described 10, 20. Bone marrow mononuclear cells were cultured in RPMI 1640 medium supplemented with 10% FBS, 20 ng/ml recombinant murine granulocyte-macrophage colony-stimulating factor (rmGM-CSF), and 20 ng/ml recombinant murine interleukin (rmIL)-4 (both from Peprotech, Inc) for 7 days. The images of immature bone marrow-derived DCs (BMDCs) were captured using an inverted light microscope (Olympus Corporation). Immature BMDCs were incubated with CD11c MicroBeads UltraPure (Miltenyi Biotec, Inc.) for 20 min at 4°C following the manufacturer's instructions. The positive fraction containing CD11c^+^ cells was analyzed by flow cytometry.

### Preparation of DRibbles and cell lysates

Autophagosome-enriched DRibbles were prepared as described previously 19. Briefly, CT-26 cells and CCSCs were treated with 100 nM Rapamycin, 100 nM Bortezomib (both from Target Molecular Corp., Boston, MA, USA), and 10 mM Ammonium chloride (Sigma-Aldrich, St Louis, MO, USA) in RPMI 1640 supplemented with 10% FBS for 16 h at 37°C with 5% CO_2_. Cells and large-cell debris were removed by centrifugation at 300 ×g for 10 min. The supernatants were then centrifuged at 10,000 ×g for 30 min at 4°C. DRibbles secreted by CT-26 cells and CCSCs were collected and adjusted to a concentration of 1 μg/μl in PBS.

CT-26 cell and CCSC lysates were prepared as previously described 21. Briefly, 3 × 10^6^ CT-26 cells and CCSCs in 1 ml PBS were lysed by five cycles of repetitive rapid freezing in liquid nitrogen and thawing in a 37°C water bath. The disrupted cells were centrifuged at 500 ×g for 30 min at 4˚C. The supernatants were collected, adjusted to a concentration of 1 μg/μl, and used as CT-26 cell- and CCSC-associated antigens.

### Cell Counting Kit-8 (CCK-8) assays

Lymphocytes derived from mice spleens were obtained as previously described 10. Briefly, different concentrations (5, 10, 20, 40 μg/ml) of CT-26 cell-derived DRibbles were incubated with lymphocytes for 72 h. To investigate DCs loaded with tumor antigens, 20 μg/ml of CCSC-derived DRibbles (SD) and lysates (SL) and CT-26 cell-derived DRibbles (TD) and lysates (TL) were incubated with the splenic lymphocytes or CD8^+^ T cells (2 × 10^6^ cells/well) for 72 h. Cell viability was assessed using a CCK-8 kit (Beyotime Institute of Biotechnology) according to the manufacturer's instructions. Cells treated with Concanavalin A (5 μg/ml) served as the positive control. Absorbance was measured at 450 nm using a Multiskan Go microplate reader (Thermo Fisher Scientific).

### Pulsing of DCs with CT-26 and CCSC extracts

CD11c^+^ DCs (1x10^6^ cells) were incubated with DRibbles (20 μg/ml) or lysates (20 μg/ml) derived from CT-26 or CCSCs in RPMI 1640 medium supplemented with 20 ng/ml rmGM-CSF and 20 ng/ml rmIL-4 for 4 h, followed by incubation with 10 ng/ml recombinant murine tumor necrosis factor-α (PeproTech) for 24 h.

### Flow cytometry

Single-cell suspension of 1 × 10^6^ CT-26 and CD44^+^ CT-26 cells (namely, CCSCs) in 95 μl of PBS was incubated with Phycoerythrin (PE)-conjugated CD44 monoclonal antibody (eBioscience, San Diego, CA, USA) in the dark for 30 min at 4˚C and subsequently analyzed using Beckman Coulter FC500 Flow Cytometer with the CellQuest Pro software (BD Biosciences, San Jose, CA, USA) to determine the number of CD44^+^ cells. Similarly, PE-conjugated CD11c monoclonal antibody (eBioscience) was used to determine the number of CD11c^+^ cells in mononuclear cells isolated from bone marrow on day 0 and immature BMDCs from day 7 of culture.

The number of CD80, CD86, MHC-I, and MHC-II cells in unpulsed DCs and DCs pulsed with DRibbles (20 μg/ml) or lysate (20 μg/ml) were determined using anti-CD86, anti-CD80, anti-MHC-I, and anti-MHC-II antibody (all from eBioscience).

The percentages of CD8^+^ IFN-γ^+^ cells in total splenic lymphocytes of each experimental and control group were determined using anti-CD8 (eBioscience) and anti-IFN-γ (Cell signaling technology, Inc., Danvers, MA, USA) antibody.

### Western blotting

CT-26 cells and CCSCs were treated with 100 nM rapamycin, 100 nM bortezomib, and 10 mM ammonium chloride in RPMI 1640 supplemented with 10% FBS for 8, 16, 24, 36, and 48 h at 37°C with 5% CO_2_. The untreated CT-26 cells and CCSCs served as negative controls. Cells collected at different time points were lysed in 1 ml radioimmunoprecipitation lysis buffer containing 10 μl phenylmethylsulfonyl fluoride. Protein concentrations were determined with a bicinchoninic acid assay kit (Beyotime Institute of Biotechnology, Co., Ltd.). The extracted proteins (50 μg each) were separated by 15% sodium dodecyl sulfate-polyacrylamide gel electrophoresis at 100 V for 1 h and then transferred to a polyvinylidene difluoride membrane at 80 V for 1 h at room temperature. The membranes were blocked with 5% bovine serum albumin for 1 h and probed with anti-LC3 (microtubule-associated protein light chain 3) antibody and anti-GAPDH antibody (both from Cell Signaling Technology, Danvers, MA, USA) overnight at 4°C. Following four washed with Tris-buffered saline containing 0.5% Tween-20, the membrane was incubated for 1 h at room temperature with horseradish peroxidase-conjugated secondary antibodies anti-rabbit IgG (Cell Signaling Technology). The protein bands were visualized by a chemiluminescence imaging system (Bio-Rad Laboratories, Hercules, CA, USA) plus Western blot detection reagents (Beyotime Institute of Biotechnology, Haimen, China).

### Transmission Electron Microscopy

After induction of autophagy for 16 h, DRibbles were harvested (10,000 ×g, 4°C, 30 min) from CCSCs and CT-26 cells. The samples were fixed in 2.5% glutaraldehyde at 4°C overnight and postfixed with 1% osmium tetroxide for 1 h at room temperature. After thorough washing with PBS, the samples were dehydrated in gradient acetone, infiltrated overnight in 1:1 acetone: Epon 812 resin (SPI Supplies, Structure Probe, West Chester, PA, USA) followed by 100% Epon 812 resin for 1 h, and embedded in the Epon 812 resin. After polymerization, 70-nm ultrathin sections were cut on an LKB-V ultramicrotome (LKB-Produkter, Bromma, Sweden) and stained with uranyl acetate and lead citrate 19. Sections were observed with a JEM-1200 transmission electron microscope (JEOL, Tokyo, Japan) and images were acquired with a charge-coupled-device CCD camera (SIS, Münster, Germany).

### Animal experiment

To establish the colon cancer model, BALB/c mice received a subcutaneous injection of 3 × 10^5^ CT-26 cells in PBS in the right flank on day 0. When the tumor size reached 100 mm^3^ on day 5, tumor-bearing mice were randomized into five treatment groups and one control group (n = 14 mice/group). Mice in treatment groups were subcutaneously injected around the tumor with a dose of 1 × 10^6^ DCs, SD-DCs, SL-DCs, TD-DCs, or TL-DCs in a total volume of 0.1 ml PBS, whereas mice in the control group received 0.1 ml PBS. Mice were vaccinated on days 5, 12, and 19 (Fig. 6A). The two perpendicular dimensions of each tumor were measured with a Vernier caliper every 3 days to calculate the tumor volume as follows: V (mm^3^) = 0.5 × a × b^2^, where a is the maximum length of the tumor, and b is the maximum transverse diameter. Mice (n = 6 mice/group) were sacrificed on day 26 and spleens were collected for further analysis. For the survival test, the time of death was recorded for animals in each group (n = 8 mice/group) to calculate the survival rate.

### CTL induction and Cytotoxicity T cell assay

Spleens were removed from mice on day 26 as described in the previous study 10. Lymphocytes were separated from the splenocytes by density gradient centrifugation using Lymphocyte Separation Medium (Beyotime Institute of Biotechnology) according to the manufacture's instruction. The lymphocytes were seeded in each well of a 96-well plate at a density of 2 × 10^6^ cells/ml in 0.1 ml RPMI 1640 medium supplemented with rmIL-2 (20 ng/ml) at 37°C with 5% CO_2_. The spleen-derived lymphocytes (immunological effector cells) from each group were examined for cytolytic activity on the target CT-26 cells in the lactate dehydrogenase (LDH) release assay using CytoTox96® Non-Radioactive Cytotoxicity Assay kit (Promega Corporation, Madison, WI, USA) following the protocol provided by the manufacturer. Briefly, CT-26 cells (2 × 10^6^ cells/ml) in a final volume of 50 μl/well in RPMI 1640 containing 10% FBS were seeded in a 96-well plate and incubated for 24 h at 37°C with 5% CO_2_. CTL (2 × 10^6^ cells/ml) were mixed to the target cells at effector-to-target (E/T) ratios of 1:1, 25:1, and 50:1 and incubated for 6 h at 37°C with 5% CO_2_. Absorbance was measured at 490 nm using a Multiskan Go microplate reader (Thermo Fisher Scientific). The percent specific cytotoxicity was calculated as [(experimental value) - (effector cell spontaneous LDH release control)] / [(target cell maximum LDH release control) - (target cells spontaneous LDH release control)] x 100. All assays were performed in triplicate.

### Statistical analyses

Data are presented as means ± standard deviation. GraphPad Prism 6.0 (GraphPad Software, CA, USA) was employed for statistical analysis. One-way ANOVA followed by a Tukey-Kramer multiple comparisons test was used to compare the corresponding data. P<0.05 was considered statistically significant. Kaplan-Meier analysis was used for survival estimations. Survival curves were analyzed by the log-rank (Mantel-Cox) test.

## Results

### Characteristics of isolated CCSCs and BMDCs

**Figure 1:**
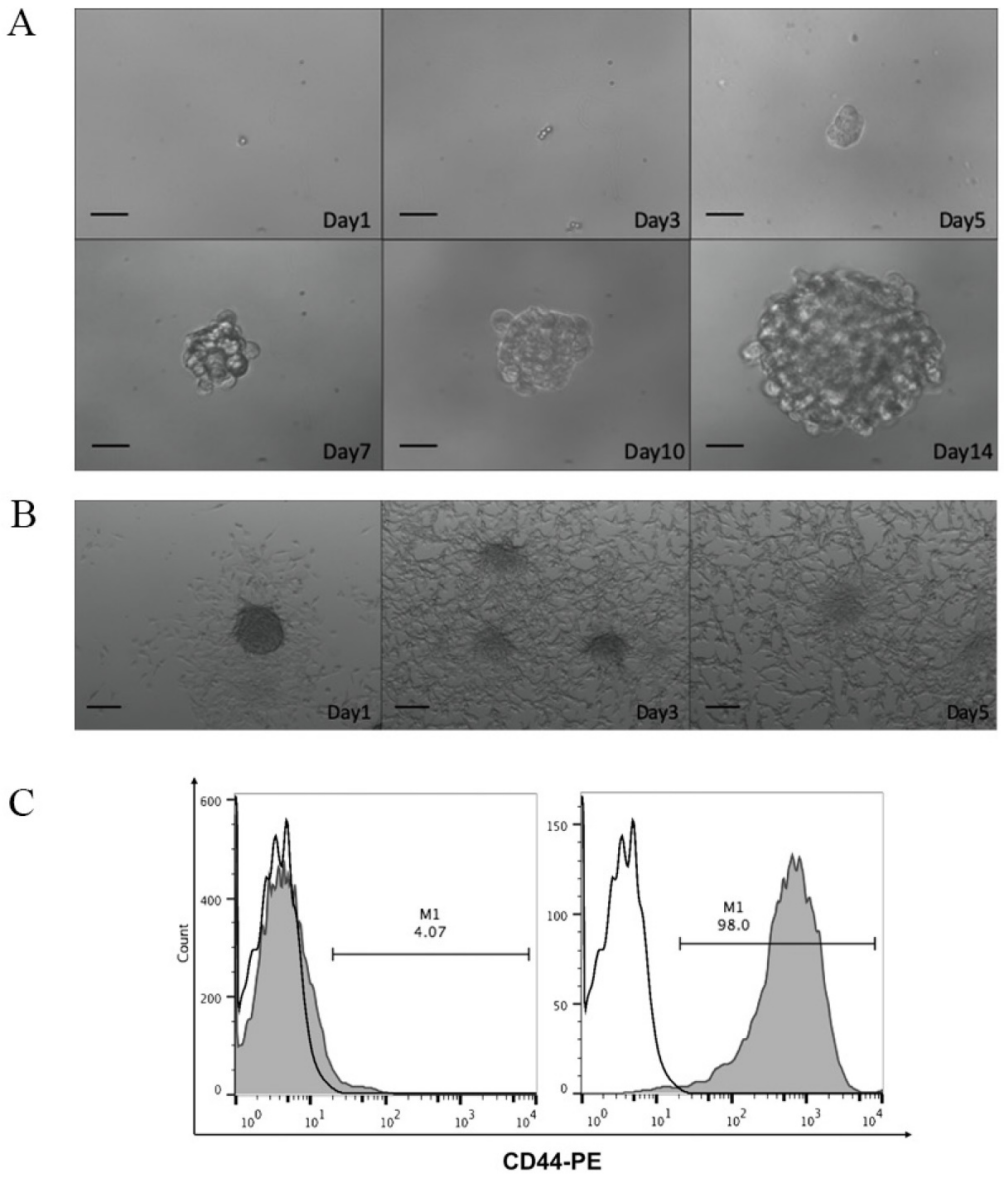
**A.** Optical micrographs presenting the morphology of CD44^+^ CT-26 cells obtained by magnetic-activated cell sorting over 14 days. Scale bar, 200 μm. **B.** Serum-induced differentiation of CD44^+^ CT-26 cells into adherent cells. Scale bar, 200 μm.** C.** CD44^+^ percentage in CT-26 cells before (left panel) and after (right panel) magnetic-activated cell sorting evaluated by flow cytometry. White, isotype control; grey, CD44^+^ expression.

To study the efficacy of CCSC-derived DRibbles as potent tumor antigens, CD44, a robust cell surface marker of CCSCs, was used to enrich CCSCs from murine CT-26 colon cancer cell line. After magnetic bead separation, the acquired CCSCs were characterized using tumorsphere-formation assay, serum-induced differentiation assay, and flow cytometric analyses of cell surface marker. In serum-free DMEM/F12 medium, a single cell suspension of the sorted CD44^+^ CT-26 cells produced sphere-forming-like cells (Fig. 1A). Clusters of sphere-forming cells appeared on day 5 and gradually increased in size through culture. These spheroid cells switched to adherent cells in the presence of 10% FBS (Fig. 1B). Flow cytometry analyses showed that high purity (98.0%) of CD44^+^ cells were obtained after MACS (Fig. 1C).

### Characteristics of BMDCs

**Figure 2:**
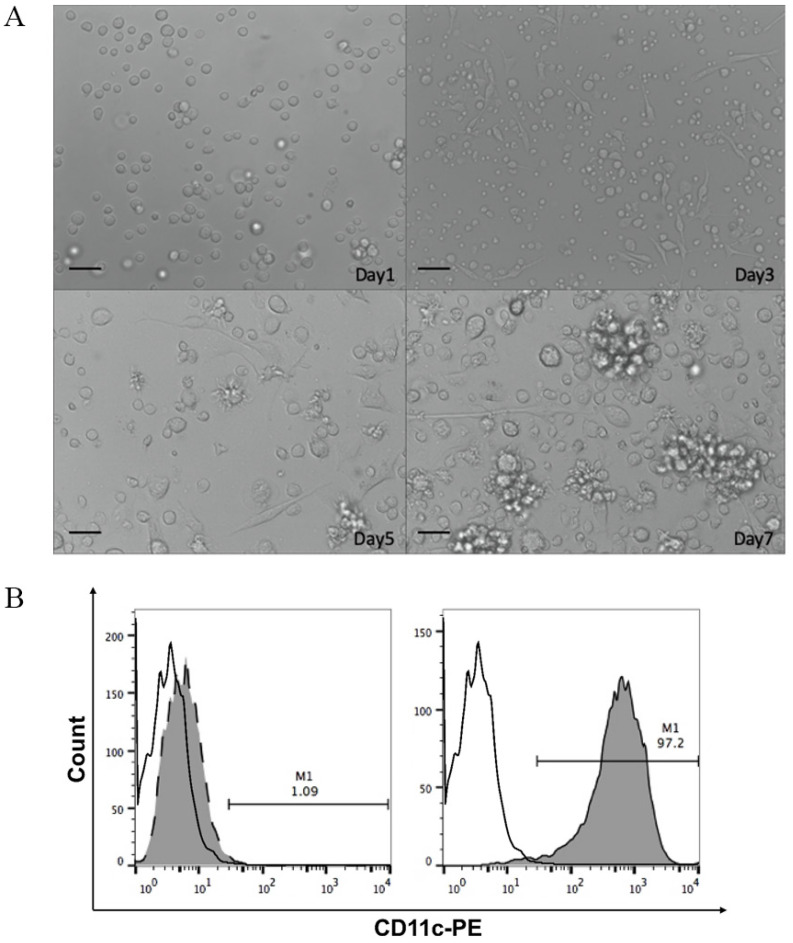
**A.** Optical micrographs presenting the morphology of dendritic cells obtained by magnetic-activated cell sorting over 7 days. Scale bar, 100 μm. **B.** The percentage of CD11c^+^ cells in mononuclear cells isolated from bone marrow on day 0 (left panel) and immature bone marrow-derived dendritic cells from day 7 of culture (right panel) evaluated by flow cytometry. White, isotype control; grey, CD11c^+^ expression.

DCs are the most effective antigen-presenting cells in the immune system. Mononuclear cells obtained from femurs and tibias of mice bone marrow were induced to differentiate into DCs using rmIL-4 and rmGM-CSF. As shown in Figure 2A, bone marrow-derived mononuclear cells gradually gathered into colonies with protrusions, branched, and extended morphology on the cell membrane after induction. The semi-adherent colonies collected on day 7 were immature DCs. DCs were further magnetically separated using anti-CD11c monoclonal antibodies. Flow cytometric analyses of surface marker CD11c^+^ showed that relatively pure (97.2%) immature DCs were obtained using the two-step separation procedure (Fig. 2B).

### Characterization of tumor-cell derived DRibbles

**Figure 3:**
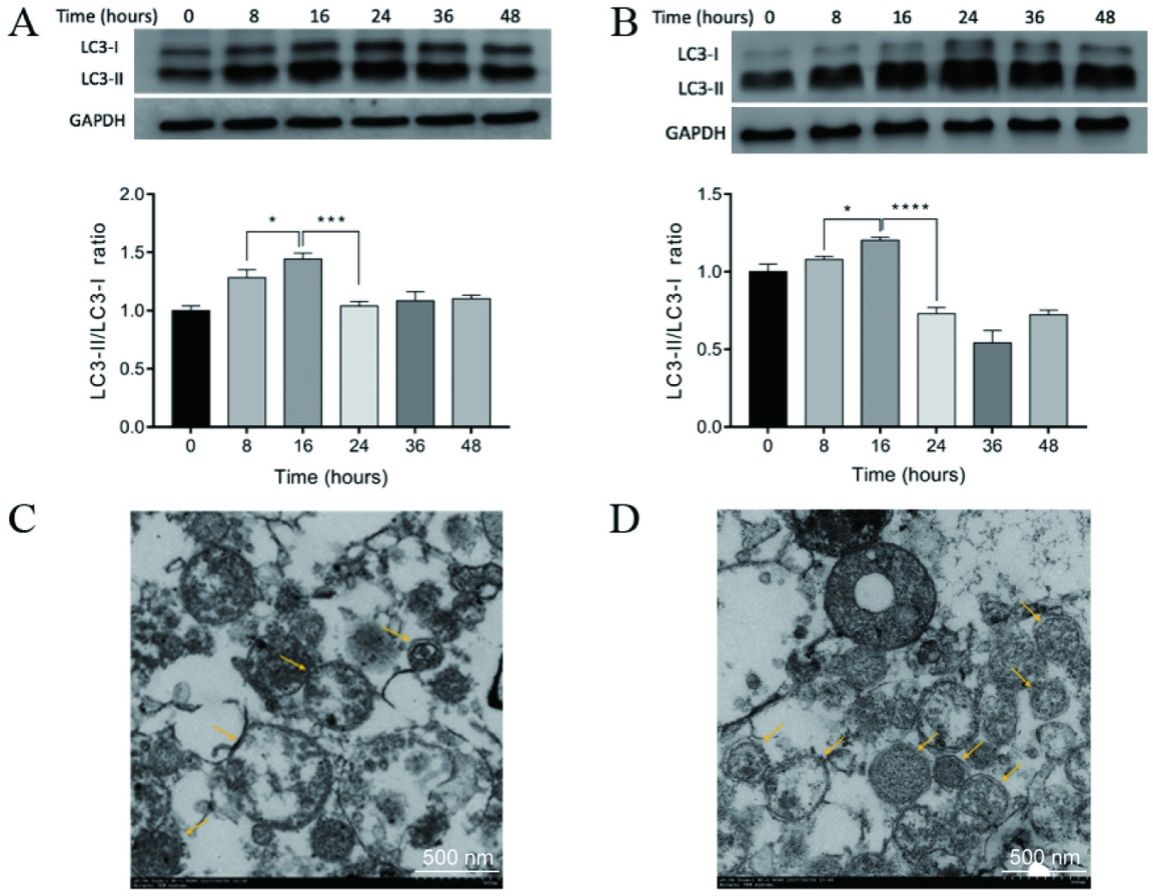
** A-B.** Autophagosomal marker LC3 detected by Western blot analysis. LC3-I to LC3-II conversion of CT-26 cells (A) or CCSCs (B) reached a maximum at 16 h after induction for autophagy. *P<0.05, ***P<0.001, and ****P<0.0001. **C-D.** A transmission electron micrograph of DRibbles in CT-26 cells (C) and CCSCs (D). The arrows show DRibbles with typical double-membrane spheroid in the ranges of 200 - 900 nm. CCSC, colon cancer stem cell; SDS-PAGE, sodium dodecyl sulfate-polyacrylamide gel electrophoresis.

Previous studies have demonstrated that the administration of rapamycin, bortezomib, and ammonium chloride induced the accumulation of autophagosomes and the formation of DRibbles in several tumor cell lines 19, 22-24. CT-26 cells and CCSCs were treated with rapamycin, bortezomib, and ammonium chloride for 8, 16, 24, 36, and 48 h. DRibbles were harvested and the levels of LC-3 (an autophagosomal marker) were analyzed using western blot. The results showed that the conversion of LC3-I to LC3-II was markedly increased in the CT-26 cells (Fig. 3A) and CCSCs (Fig. 3B) in a time-dependent manner and reached a maximum at 16 h following the induction of autophagy. Therefore, an induction time of 16 h was used in the following experiments.

The ultrastructure of DRibbles was examined under transmission electron microscopy. Numerous vesicles were characterized by a unique double-membrane structure containing defective organelles and were 200-900 nm in diameter, suggesting that DRibbles were obtained after induction of autophagy in CT-26 cells (Fig. 3C) and CCSCs (Fig. 3D).

### DRibbles promote lymphocyte proliferation

**Figure 4:**
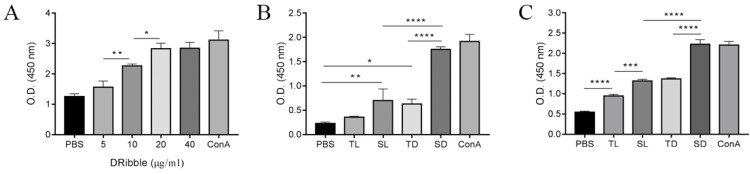
**A-C.** CCSC-derived DRibbles promoted lymphocytes proliferation analyzed using CCK-8 assays. (A-B) mouse splenic lymphocyte proliferation. (C) CD8^+^ T cell proliferation. PBS and ConA (5 μg/ml) served as a negative and positive control, respectively. *P<0.05, **P<0.01, and ****P<0.0001. TL, CT-26 cell lysates; SL, CCSC lysates; TD, CT-26 cell DRibbles; SD, CCSC DRibbles; ConA, Concanavalin A; CCSC, colon cancer stem cell; PBS, phosphate-buffered saline.

To investigate the optimal concentration of DRibbles on lymphocyte viability, DRibbles of various concentrations (5, 10, 20, and 40 μg/ml) were incubated for 72 h with splenic lymphocytes and the lymphocyte viabilities were analyzed using a CCK-8 assay. The results showed that DRibbles induced the viability of lymphocytes in a dose-dependent manner and the proliferation of lymphocytes reached a maximum when incubated with 20 μg/ml DRibbles (Fig. 4A). Thus, 20 μg/ml DRibbles was used as the optimized concentration in the following experiments.

Next, DRibbles (20 μg/ml) and lysates (20 μg/ml) derived from either CT-26 or CCSCs were incubated with splenic lymphocytes and CD8^+^ T cells for 72 h to evaluate their effect on lymphocyte viability. The results showed increased lymphocyte proliferation in SD, TD, and SL compared with the control group. Moreover, SD has the strongest capability in inducing lymphocyte proliferation *in vitro* (P<0.0001) among other treatment groups and the negative control group (Fig. 4B). In particular, SD stimulated the proliferation of CD8^+^ T cells more efficiently compared to other experimental and control groups (P<0.001), suggesting that SD were effective antigens for cross-presentation (Fig. 4C).

### DRibbles derived from CCSCs induced higher expression of surface markers on DCs

**Figure 5:**
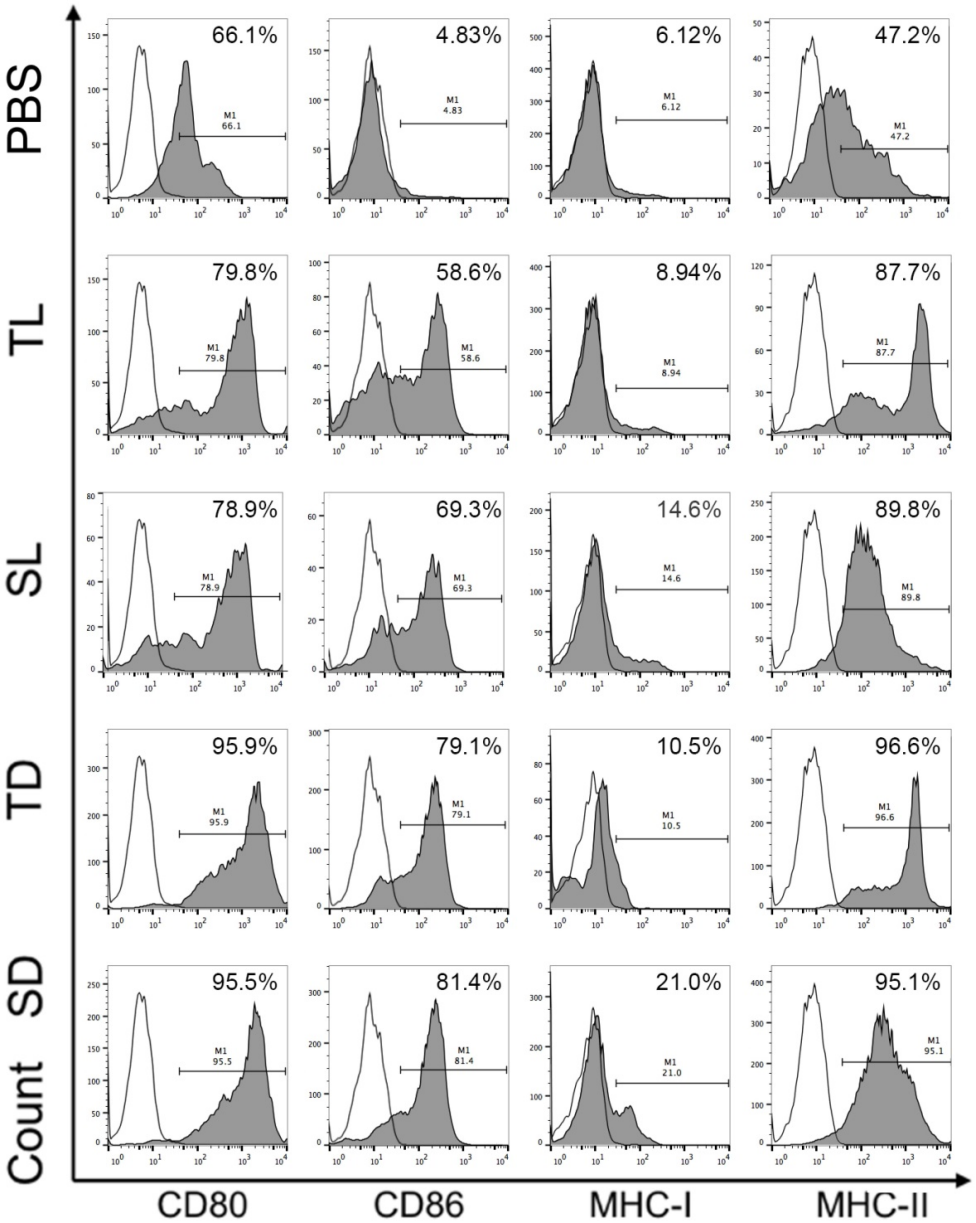
Surface molecules of DCs loaded with DRibbles or lysates analyzed by flow cytometry. CD11c was used as the maker to gate DCs. CCSC, colon cancer stem cell; DC, dendritic cell; TL, CT-26 cell lysates; SL, CCSC lysates; TD, CT-26 cell DRibbles; SD, CCSC DRibbles; PBS, phosphate-buffered saline. White, isotype control.

To investigate the efficacy of DRibbles in modulating DC function, DRibbles and lysates derived from CCSCs or CT-26 cells were incubated with the immature DCs for 24 h and the expression of surface marker CD80, CD86, MHC-I, and MHC-II on DCs were analyzed using flow cytometry. As shown in Figure 5, SD and TD induced upregulation of CD80, CD86, and MHC-II on DCs compared to SL and TL. Particularly, SD induced upregulation of MHC-I molecules. The expression levels of CD80 and CD86 represent DC maturation and are related to the ability of DCs to provide a secondary signal to stimulate the activation of T cells, while the expression levels of MHC-I and MHC-II molecules are closely associated with the DC capability in presenting antigens. Therefore, our result suggested that DRibbles were more effective than lysates in inducing DC maturation, and in particular, SD has the strongest ability to present antigens to DCs by upregulating MHC-I.

### DRibbles induce antitumor immunity in tumor-bearing mice

**Figure 6:**
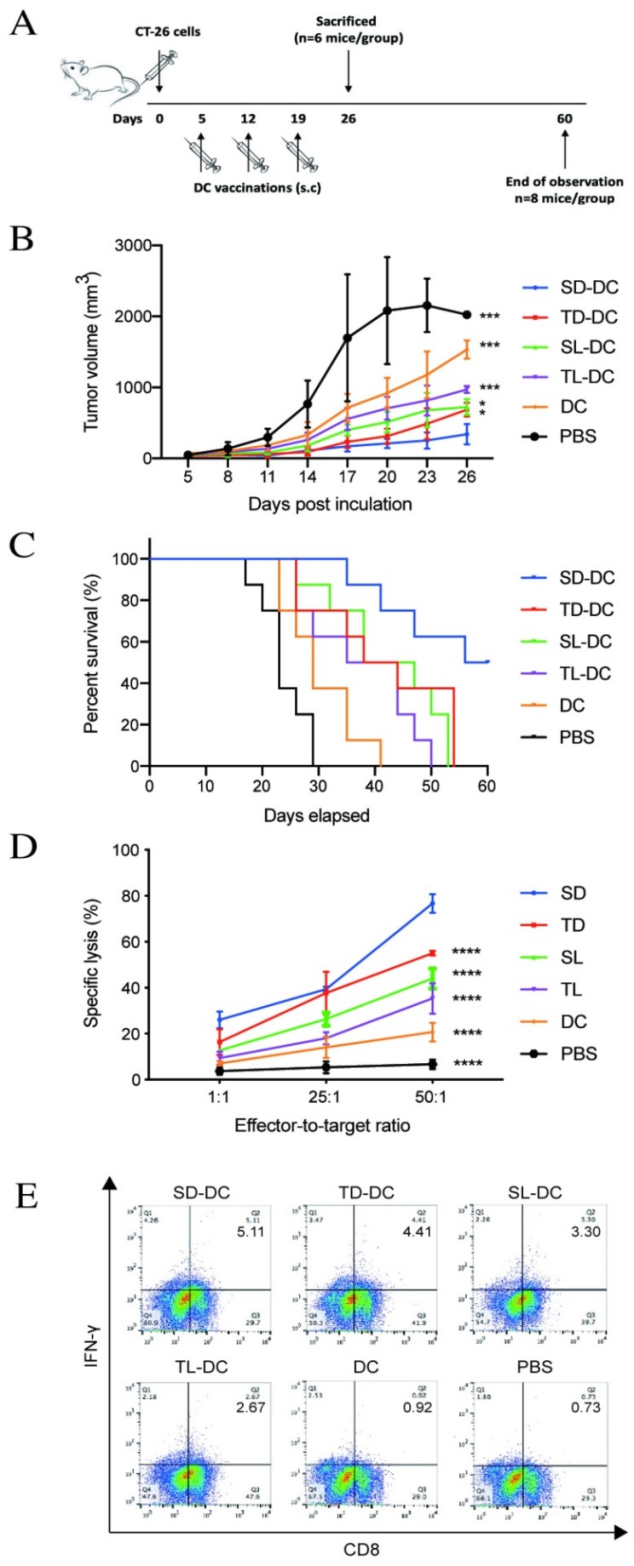
** A.** Vaccine scheme in an established murine colon cancer model of colon carcinoma. **B.** Average tumor volume (n=6 mice/group). **C.** Percentage of survival (n=8 mice/group). **D.** Cytotoxic T lymphocyte-stimulated lysis of CT-26 cells. **E.** Percent of IFN-γ^+^ CD8^+^ T cells analyzed by flow cytometry. *P<0.05, ***P<0.001, ****P<0.0001 vs. SD-DC group. DC, dendritic cell; SD-DC, DC loaded with CCSC-derived DRibbles; SL-DC, DC loaded with CCSC-derived lysates; TD-DC, DC loaded with CT-26 cell-derived DRibbles; TL-DC, DC loaded with CT-26 cell-derived lysates; PBS, phosphate-buffered saline; CCSC, colon cancer stem cell; IFN-γ, interferon-gamma.

To investigate the anti-tumor efficacy of DRibbles *in vivo*, vaccines of DCs loaded with either DRibbles (SD and TD) or lysates (SL and TL) derived from CT-26 cells or CCSCs were subcutaneous injected into tumor-bearing mice. On day 26, the group of mice immunized with SD-DCs presented the smallest tumor size (Fig. 6B) and improved survival (Fig. 6C) compared to other treatment groups and the control group. Subsequently, splenic lymphocytes from DC-treated mice were tested for cytotoxicity using an LDH assay. Pulsed DC treatments induced potent antitumor immunity and tumor-specific cytotoxicity *in vitro* (Fig. 6D). Immunization of tumor-bearing mice with SD-DCs induced significantly stronger cytolytic activity against CT-26 cells at E/T ratio of 1:50 (P<0.0001). The percent cell lysis in SD-DC group was 76.67 ± 4.04%, which was much higher than in the TD-DCs (55.03 ± 1.04%), SL-DC (44.00 ± 4.36%), TL-DC (35.33 ± 6.66%), and DC (20.67 ± 4.04%), and PBS (15.33 ± 2.31%) groups (P<0.0001 for all comparisons). Furthermore, the flow cytometry analyses showed that the percentages of IFN-γ producing CD8^+^ T-cells were increased in SD-DC group compare with the other groups, demonstrating that SD-DC vaccine may facilitate the antigen cross-presentation to CD8^+^ T cells to produce an effective anti-tumor immune response. Taken together, these findings showed that the vaccination with SD-DCs inhibited tumor growth, prolonged survival, and induced antigen-specific T cell responses more efficiently than TD-DCs, lysates-loaded DC, DC, and PBS groups in a CT-26 murine colon cancer model.

## Discussion

DC-based anti-tumor vaccines have great potential for cancer treatment. To date, a variety of cancer vaccines have been conducted in clinical trials, highlighting new therapeutic approaches to advance the patients' immunity 25. However, these vaccines had several drawbacks, such as poor cross-presentation of TAAs to antigen-presenting cells that resulted in weak immunogenicity, immune suppression, and immune escape due to antigen loss, which are critical for the success of cancer vaccines. Hence, the development of DC-based vaccines capable of enhancing cross-presentation in priming antitumor T-cell responses is of great interest.

Types of vaccines are varied by the composition of DC-pulsed antigens including tumor lysates 7, RNAs 9, and tumor-derived peptides 26. In recent years, tumor-cell derived autophagosomes (DRibbles), which enriched in long-lived proteins, short-lived proteins, and DRiPs after inhibition of proteasome and lysosome function, has demonstrated efficient cross-priming antigen-specific naïve CD4^+^ and CD8^+^ T-cells *in vitro* and *in vivo*; and DRibbles-based DC vaccine has shown high efficiency in inducing antitumor immune responses in immunodeficient mice models 16, 23, 27, 28. In our study, DRibbles were isolated from mouse colon carcinoma cell line CT-26 and CD44^+^ CCSCs by augmenting autophagy and inhibiting protein degradation with the treatment of rapamycin, bortezomib, and ammonium chloride. The expression of the typical autophagosomal marker, LC3, was detected by western blot and the results revealed that the LC3-I to LC-II conversion reached a maximum after 16 h of autophagy induction in both cells. The acquired DRibbles were vesicles in a spherical shape with double-membrane structure in a range of 200 - 900 nm in diameter, as evidenced by transmission electron microscopy.

DCs are professional and excellent antigen-presenting cells, making them a great tool to study cross-presentation. The study of cross-presentation necessitates a large number of DCs, but the relative rarity of DCs in circulation and in secondary lymphoid tissues makes it a big challenge to isolate sufficient numbers of cells for *in-vitro* experiments. However, Inaba *et al* have shown the protocol of how to differentiate bone marrow progenitors into DCs* in vitro* for cross-presentation experiments 20. In our study, we used BMDCs because they are generally used as antigen-presenting cells in numerous DC-based immunotherapy studies and BMDCs have been shown to use both the cytosolic and vacuolar pathways for cross-presentation, while CD8α^+^ subpopulation of classical conventional DCs isolated from secondary lymphoid tissues only utilizes the cytosolic pathway^20^, ^24^, ^29^-^33^. In mice, BMDCs, lymphoid organ-resident CD8^+^ DCs and migratory CD103^+^ DCs are efficient at cross-priming antigen for MHC class I presentation 31-33.

To determine the efficacy of DRibbles and lysates derived from CCSCs and non-CCSCs in stimulating naïve T-cells, DRibbles and lysates were incubated with mouse splenic lymphocytes and CD8^+^ T cells *in vitro* and the cell proliferation was examined using a CCK-8 assay. Our results revealed a more potent stimulation of lymphocyte proliferation by CCSCs-derived DRibbles than any other groups. Furthermore, by incubating immature BMDCs with DRibbles and lysates, CCSC-derived DRibbles significantly induced the upregulation of MHC-I molecules compared to other groups, suggesting a stronger capability of CCSC-derived DRibbles in inducing efficient cross-priming of tumor-specific CTLs and activating naïve CD8^+^ T cells. However, DRibbles were only slightly more potent than the lysates in inducing the expression of MHC-II molecules. DRibbles also efficiently induced upregulation of CD80 and CD86 compared to lysates and negative control. CD80 and CD86 are B7 molecules that interact with the costimulatory molecule CD28 expressed by T cells for initiating primary T cell response 34. CD80 may be more efficient in inducing anti-tumoral responses, whereas CD86 preferentially induces the production of a Th2 response 35, 36. Our results were consistent with other studies that CD86 and MHC-I molecules on DCs were significantly upregulated, while the upregulation of MHC-II was not evident 19. DRibbles were superior to lysates as efficient antigen carriers may partially because DRibbles incorporated short-lived proteins and DRiPs, which degraded rapidly by proteasome and lysosome pathways and are not efficiently cross-presented by DCs under normal conditions. After inhibition of proteasome- and lysosome-mediated degradation, short-lived proteins and DRiPs became readily available for cross-presentation as they were shunted into autophagosomes. Thus, DRibbles contain most of the peptides presented on MHC-I molecules for cross-priming antigen-specific CD8^+^ T cells, which may be beneficial to improve the efficiency of tumor recognition and destruction 17, 23.

In our murine model, mice vaccinated with SD-DCs exhibited high immunogenicity compared to those vaccinated with TD-DCs, lysates-pulsed and unpulsed DCs, and PBS, suggesting that CCSC-derived DRibbles were more immunogenic compared to other treatment groups and the control group. Our findings were consistent with the results in several murine carcinoma models, including head and neck cancer and oral squamous cell carcinoma, that more significant suppression of tumor growth and life prolongation was observed in the DRibble-DC vaccinated group compared to lysate-DC vaccinated group 19, 24. Moreover, our *in-vitro* assays revealed the efficacy of CCSC-derived DRibbles in inducing anti-tumor immune responses, as demonstrated by a significant greater cytolytic activity and higher percentages of IFN-γ^+^-producing CD8^+^ T-cells in the SD-DC vaccination group.

## Conclusions

In this study, CCSCs isolated from mouse colon carcinoma cell line CT-26 were treated with rapamycin, bortezomib, and ammonium chloride to induce autophagy and form DRibbles. CCSC-derived DRibbles significantly enhanced DC maturation and strengthen the immunostimulatory function of DCs. In the tumor-bearing mice model for colorectal carcinoma, DC vaccines loaded with CCSC-derived DRibbles inhibited tumor growth, improved survival, and induced CTL responses more efficiently compared with the DCs loaded with or without tumor lysates. Although more experiments may be necessary to demonstrate the underlying mechanisms of the increase in cross-presentation by CCSC-derived DRibbles, our preliminary findings suggest the efficiency of novel immunotherapeutic anti-tumor approaches based on DRibbles derived from CCSCs in colorectal cancer treatment.
